# Some Interesting Post-mortems

**Published:** 1895-03

**Authors:** A. G. Alverson

**Affiliations:** Bloomington, Ill.


### SOME INTERESTING POST-MORTEMS.

BY A. G. ALVERSON, V.S.

Case I.—I was called one evening about five o'clock to see a large gray gelding which had come into the owner's possession the day previous. The animal showed some slight pain, evidenced by kicking or stamping with the hind feet and switching the tail. Pulse, respiration, and temperature nearly normal. There was a dirty, gummy appearance of the hair down the back part of front legs and along the median line of belly to within about twelve inches of the sheath, where the hair was nearly gone and the skin somewhat excoriated. This led to an examination of the penis. This was found to be swollen at the lower end, with tenderness at the opening of the urethra. Rectal exploration demonstrated that the bladder was only partially filled with urine. Diagnosed obstruction of urethra, which could only be permanently relieved by operation.

Saw no urgent necessity for catheterization, as the urine was dribbling away constantly and the bladder not distended.

The owner concluded to ship the horse and sell him for whatever he could get. The next morning the animal was sent to the infirmary in a dying condition.

I tried, however, to pass the catheter and found the urethra plugged with a deposit of lime salts. Removed considerable of the deposit as far as in reach and passed a uterine sound (metal) to pave the way, so to speak, and then succeeded in passing the catheter, but no urine came. Animal died in a short time and autopsy was made the next morning.

*Autopsy.* A little more than a finger length up the urethra was a large mass of lime-salt deposit. The bladder, nearly black in color, had numerous adhesions to the pelvis and pelvic viscera.

There was a hole two or three inches in circumference through the wall and internally the wall was studded with this sabulous deposit.

Case II.—This patient, a two-year-old grade draught gelding, standing in a box stall, showed on the morning of October 14th marked symptoms of depression, temperature below the normal, pulse 40, head held low, eyes dull, and breathing deep and slow. A very fetid odor escaped from the mouth. Diagnosed brain trouble; probably reflex, from indigestion.

The animal was bled, given a dose of linseed oil, and ordered salicylate of soda with stimulants, and a second dose of oil in the evening. The animal died the next forenoon.

*Autopsy soon after death.* Found stomach distended with food in a semi-dry condition. Cœliac axis was the seat of an aneurism about the size of a man's head. Several portions of the bowels were adherent to its surface, including the duodenum which, at point of union, was less than half its natural capacity. This constriction of the duodenum seemed to me to be the cause of the stomach and brain symptoms, by not allowing the ingesta to pass on. The bowels were without inflammation throughout.

Case III.—On the morning of May 10, 1891, I went some eight miles in the country to see a sick mare. Reached the place about eight o'clock. The patient was a four-year old gray mare, with a healthy foal a few weeks old by her side.

History given was that the young horses on the place had all been more or less affected with strangles. About six weeks before time of visit, this mare was not very well, but neither swollen in any perceptible place, nor was there any discharge from the nose.

She had not thrived well since that time, although had given birth to the healthy foal.

Two or three times she had shown slight attacks of colic, which soon passed off without receiving any particular treatment. This attack came on about eleven in the evening of the day previous to my visit, and she had been very uneasy since early in the evening. Examination showed a scarcely perceptible pulse; temperature, 102°; anxious expression, with slight tremors over the body, particularly at the shoulders; head occasionally extended and upper lip turned up; a sour, foul-smelling breath, and very little, if any, peristaltic action. Diagnosis, inflammation of the stomach and bowels.

Gave sodas, stimulants, and fluid ext. cannabis indica, but the animal grew rapidly worse and died about twelve hours from the time this attack came on. The post-mortem, made immediately after death, showed a ruptured stomach, with abdominal cavity filled with water and undigested food. Further examination showed a mesenteric abscess larger than a man's head containing quite a quantity of creamy pus.

Both large and small intestines were adherent to the surface of abscesses. Here was the primary cause for the condition of stomach and bowels.

CASE IV.—In May or June of last year I went with Dr. Williams, my former partner, to see the subject of the following article. The cow had at that time been ailing for several months, and was found lying in the stable-yard, looking emaciated and breathing laboriously. Was with some difficulty persuaded to stand. Poor, hide-bound, enlarged in joints, and showing symptoms which at once led to an examination of the lungs. Both were dull in patches aggregating more than half the surface.

Could obtain no history of contagion, but we considered the case must be one of tuberculosis, and gave that as our opinion, advising destruction, which was not done for two or three weeks, during which time she grew gradually worse.

*Autopsy.* Substance of the lungs was more like the consistency of kidney, lobulated; but the different parts showed great variations in density. Some were entirely devoid of air, some containing a little, while other parts had been semi-active until death. The bicuspid valves had a honey-combed appearance, and must have been incapable of performing their function. The walls of the heart were normal.

Was not the forcing back of the blood from the heart through the pulmonary veins the cause of the condition of the lungs?

BLOOMINGTON, ILL.

---

The most earnest, conscientious, and determined opposition is being developed against the direction and control of infectious and contagious diseases being left in the hands of any one man, especially a layman, lest the arbitrary power thus lodged might be used against the best interests of the State, and an unsatisfactory solution of at least one very important subject at this time—tuberculosis.

